# Health economics of health justice partnerships: A rapid review of the economic returns to society of promoting access to legal advice

**DOI:** 10.3389/fpubh.2022.1009964

**Published:** 2022-11-15

**Authors:** Rachel Granger, Hazel Genn, Rhiannon Tudor Edwards

**Affiliations:** ^1^Centre for Health Economics and Medicines Evaluation, Bangor University, Bangor, United Kingdom; ^2^Faculty of Laws, University College London, London, United Kingdom

**Keywords:** health justice partnerships (HJP), health economics, welfare legal advice, return on investment (ROI), rapid review, cost-consequence analysis (CCA), cost-utility analysis (CUA), public health

## Abstract

**Background:**

Welfare legal problems and inadequate access to support services follow both the socioeconomic and the health inequalities gradients. Health Justice Partnership (HJP) is an international practitioner-led movement which brings together legal and healthcare professionals to address the root causes of ill health from negative social determinants. The aim of this paper was to identify the current evidence base for the cost-effectiveness of HJP or comparable welfare advice services.

**Methods:**

A rapid review format was used, with a literature search of PubMed, CINAHL, ASSIA, PsycINFO, Medline, Cochrane Library, Global Health and Web of Science identifying 496 articles. After removal of duplicates, 176 papers were screened on titles and abstracts, and 20 papers met the eligibility criteria. Following a full-text screening, a further 14 papers were excluded due to lack of economic evaluations. Excluded papers' reference lists were scanned, with a further 3 further papers identified which met the inclusion criteria. A final pool of nine studies were included in this review.

**Results:**

Studies focused on the financial benefit to service users, with only three studies reporting on cost effectiveness of the interventions. Only one study reported on the economic impact of change of health in service users and one study reported on changes in health service use.

**Conclusion:**

This review highlights the current evidence gap in evaluating the cost-effectiveness of adequate access to free legal welfare advice and representation. We propose that an interdisciplinary research agenda between health economics and legal-health services is required to address this research gap.

## Introduction

Socioeconomic deprivation is acknowledged to cast a long shadow on the health and wellbeing of affected populations, not only within but across generations (1). We are familiar with models of the socioeconomic determinants of health e.g., the Dahlgren-Whitehead model (2). The 2010 Marmot review (3) proposed six policy objectives required to reduce health inequalities:

1. Give every child the best start in life
2. Enable all children, young people and adults to maximize their capabilities and have control over their lives
3. Create fair employment and good work for all
4. Ensure a healthy standard of living for all
5. Create and develop healthy and sustainable places and communities
6. Strengthen the role and impact of ill-health prevention.

Successful and sustainable approaches to reducing these health inequalities will depend upon stronger collaborative working across sectors.

Experience of welfare legal problems and inadequate access to free legal support follows not only the socioeconomic gradient, but also the health inequalities gradient (4–7). Although legal issues are embedded in most social determinants of health and are recognized at the macro legislative level, the need for free legal services to improve health at a local or individual level has largely been overlooked. It was not specifically highlighted in the 10-year reviews by Marmot and colleagues (8, 9), nor in the current discourse on “leveling up” (10).

It is acknowledged that the root cause of a significant proportion of healthcare usage (in both primary and secondary healthcare) in socioeconomically disadvantaged populations is not only due to the direct health outcomes associated with depression but associated “non-health” socio-legal issues, such as family breakdown, employment problems, access to welfare benefits and inadequate housing (11). People on low income or experiencing poor physical or mental health are more likely to have a need for legal support in accessing welfare benefits, managing debt or addressing housing issues. However, they often have difficulty accessing legal support and frequently approach inappropriate sources, such as GPs who are unlikely to be able to provide guidance (12, 13). This additional strain on GPs in deprived areas is not only unlikely to be effective for the individual but it deflects primary care resources away from healthcare provision in already overburdened GP practices.

Health Justice Partnership (HJP) is an international practitioner-led movement which brings legal and healthcare professionals together to address the root causes of ill health among low income and vulnerable groups from negative social determinants (4). HJPs, which are also referred to as medical-legal partnerships (MLP) in the US, are collaborations between health and free welfare legal services providing advice and support for patients experiencing health-harming challenges such as housing problems (e.g., landlord/tenant disputes, housing discrimination), benefits (e.g., benefits accessibility and claims denials), family (e.g., child support and civil protective orders) and consumer (e.g., bankruptcy and utility shut-offs). Welfare legal service providers working in partnership with health services are therefore highly relevant to public health, as they focus on prevention, by addressing upstream systematic social and legal problems that affect patient and population health. Many of the health issues experienced by individuals are due to, or exacerbated by, the effect of unenforced laws or incorrect denial of critical services or support that they are entitled to. Rather than creating new laws, the primary aim of HJP is to ensure that public bodies comply with current statutory responsibilities and that individuals receive the benefits and conditions to which they are legally entitled.

The aim of this paper was to present a rapid literature review to identify the current evidence base for the cost-effectiveness of HJP or comparable welfare advice services. In addition, to investigate if there is evidence for the impact of these services on wider measures, such as service user health and healthcare usage.

## Methods

### Search strategy

The literature search strategy followed rapid review procedure (14). The period 1st January 1995 to 14th July 2022 used covers the period since HJP were first reported (15). The following academic databases were examined: PubMed, CINAHL, ASSIA, PsycINFO, Medline, Cochrane Library, Global Health and Web of Science. Titles and abstracts were searched, with search terms based on the criteria of “social welfare legal advice” AND “health economic evaluation methods”. The full list of terms searched for were: “legal support” OR “legal advice” OR “legal service” OR “health justice” OR “health justice partnership” OR “health-justice partnership” OR “medical legal partnerships” OR “medical-legal partnerships” OR “health law partnership” OR “Citizens Advice” OR “CAB” OR “welfare legal advice” OR “ welfare benefits” OR “welfare claims” OR “debt advice” OR “housing advice” OR “immigration advice” OR “family advice” AND “cost-benefit analysis” OR “cost-utility analysis” OR “cost-effective analysis” OR “cost effective^*^” OR “social return on investment” OR “return on investment” OR “social cost-benefit analysis” OR “ cost-minimization analysis” OR “cost-consequence analysis” An additional search in Google Scholar was also conducted using the same search terms.

### Search inclusion

**Figure 1:**
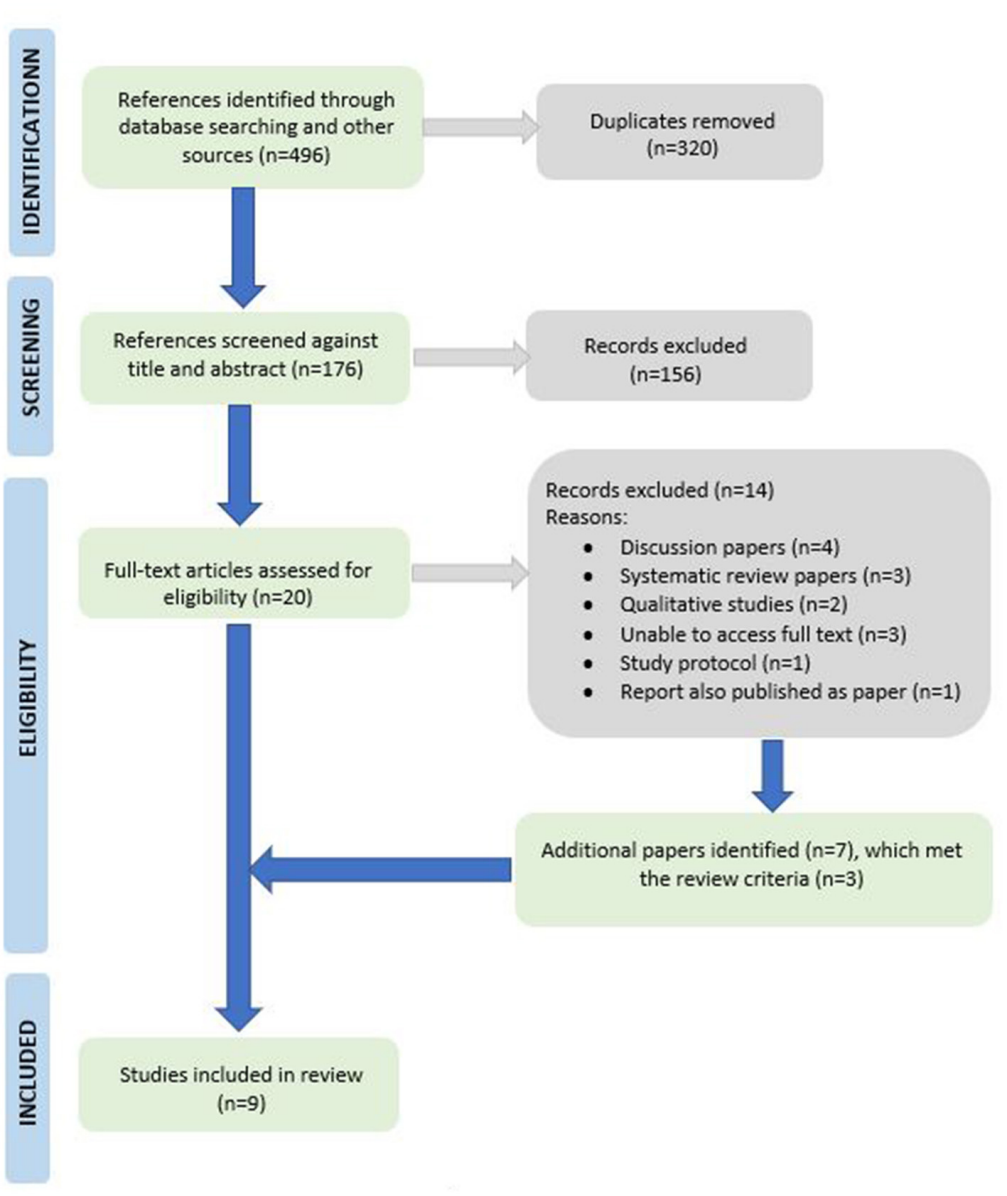
PRISMA study selection flowchart (25).

The database search identified 496 references. After the removal of duplicates, 176 references were screened against title and abstracts, with a further 156 papers being excluded. This left a total of 20 papers to be assessed on their full text articles. All searches and article assessments were carried out by the lead author (RG). Papers that did not contain financial or economic analyses were excluded. Excluded papers included systematic reviews, discussion papers, qualitative studies, a trial protocol paper and a report that was later published in a peer reviewed journal. Although reviews and discussion papers were not included, their reference lists were scanned to identify additional citations. This identified seven additional papers that were retrieved for further examination, three of which met the inclusion criteria for this review. A final pool of nine articles was included in this rapid review (see Table 1). Figure 1 shows the PRISMA flow diagram for this study. Quality assessments were carried out for the nine included articles using the Joanna Briggs Institute JBI critical appraisal tools for Case Reports, Randomized Controlled Trials, Quasi-Experimental and Economic Evaluation.

**Table 1:** Summary of included studies.

**References, (country)**	**Study details**	**Participants and setting**	**Key findings**
Teuful et al. (16), US	**Study design:** Case study, mixed methods **Type of Intervention:** Civil Legal Aid service provided for women experiencing intermate partner violence (IPV). **Data collection methods/outcomes measured:** SROI, changes in private and public benefits income over a 12-months **Quality rating:** medium	**Sample size:** *n* = 82 **Participants and setting:** Adult women currently experiencing IPV and who have accessed legal aid support from Iowa Legal Aid, US. **Dates of data collection:** Service users in 2015-16	**Primary findings:** Reported increase of income of service users of $2.41 for every $1 invested in the services. The average total income increase was $5,500 per service user, which was driven by private rather than welfare income. **Additional findings:** The odds for being in poverty decreased a year after using the service- approx. 2.5 times lower (rOR = 0.28) than before service use.
Citizens Advice 2015/16 Report (17), UK	**Study design:** Case study **Type of Intervention:** Standard CA welfare advice provided in CA centers. **Data collection methods/outcomes measured:** ROI and users' and volunteers' evaluation (qualitative) of the CA service **Quality rating:** medium	**Sample size:** *n* = 4,200 (*n* = 2,700 service users, *n* = 1,500 CA volunteers). **Participants and setting:** Adults using the CA welfare advice service or adults working as CA volunteers in the UK. **Dates of data collection:** Services users and CA volunteers in the financial year 2015/16	**Primary findings:** For every £1 invested a total of £20.57 was generated, consisting of £10.97 financial benefits to service users, £1.52 of savings to government, and an estimated £8.08 in wider economic and social benefits (including participating and productivity for clients and volunteers). **Additional findings:** 65–75% of clients stated that welfare issues were causing them stress and anxiety, although it was not reported how service usage changed these levels
Woodhead et al. (18), UK	**Study design:** prospective quasi-experimental controlled trial **Type of Intervention:** Provision of CA welfare advice in GP surgeries. **Data collection methods/outcomes measured:** financial impact including ROI; proportion meeting criteria of common mental disorder using General Health Questionnaire and SWEMWBS scales; impact on health and social care utilization; changes in meeting criteria for common mental health disorders. Financial measurements were taken over 8 months, all others over 3 months. **Quality rating:** High	**Sample size:** *n* = 816 (*n* = 278 intervention and *n* = 613 control) **Participants and setting:** Adults using CA debt advice service in GP settings, UK. **Dates of data collection:** Dec. 2015–Dect 2016	**Primary findings:** ROI of additional income of £15 to service users per £1 funder investment. Service users increased welfare income by an average of £2,689 during 8-month study period. Common mental disorders reduced among women (rOR = 0.37) and black service users (rOR = 0.09) compared to controls. No changes were reported for consultation rates at 3 months. **Additional findings:** Service users with a positive financial outcome reported improved wellbeing and reductions in financial strain (rOR = 0.42).
Gabby et al. (19), UK	**Study design:** Pilot RCT, mixed methods, including economic analysis **Type of Intervention:** Provision of CA welfare advice in GP surgeries in England and Wales. **Data collection methods/outcomes measured:** trial feasibility, changes in depression score (Beck Depression Inventory) and Anxiety (Back Anxiety Inventory) over 4 months of trial, economic analysis planned, including HRQoL (using EQ-5D-5L) and SWEMWEBS and healthcare usage measures. **Quality rating:** High	**Sample size:** *n* = 61 (*n* = 32 intervention and *n* = 29 control). **Participants and setting:** adults using primary care services with mild-moderate depression (using Beck depression score) and debt worries, UK. **Dates of data collection:** July 2014–Aug 2015	**Primary findings:** Due to dropout rates, data was insufficient to carry out proposed economic evaluations. **Additional findings:** Mean depression scores and anxiety scores improved in the service users compared to the control group in the descriptive statistics but were not statistically significant. Descriptive statistics showed lower levels of acute healthcare usage and higher levels of community healthcare service usage for service users.
Caiels and Thurston (20), UK	**Study design:** Case study, mixed methods **Type of Intervention:** Provision of CA advice in GP surgeries. Referral by healthcare professionals or self-referral. **Data collection methods/outcomes measured:** Improvements of financial situation of service users; changes in anxiety and general health (SF-12 questionnaire); participants and providers experience of the service. **Quality rating:** Low	**Sample size:** *n* = 333 service users (*n* = 81 participated in health measures and qualitative study). **Participants and setting:** Adults using welfare advice services in primary healthcare settings in Warrington, UK. **Dates of data collection:** Aug 2003–Sept. 2004	**Primary findings:** Total financial improvement was £346,754 (mean average of £1,041 per participant), which was due to increased welfare benefits and debt written off. **Additional findings:** 68% reported feeling less anxious after using the service, but there was no significant difference in the physical or mental health components of the SF-12 scores.
Naven et al. (21), UK	**Study design:** Case Study, mixed methods **Type of Intervention:** Money Advice provided welfare advice services in child health settings. Referral by healthcare professionals. **Data collection methods/outcomes measured:** financial improvement to service users, service users and healthcare professionals experience, factors affecting implementation. **Quality rating:** Low	**Sample size:** *n* = 2,516 **Participants and setting:** pregnant woman and families with children under 5 in the Greater Glasgow area, UK. **Dates of data collection:** Oct. 2010–March 2012	**Primary findings:** 1 in 2 service users were entitled to additional financial support and the average gain per service user was £3,404. **Additional findings:** Service users reported a reduction in stress and an improvement in mood, security, self-worth and relationships with family and friends.
Moffatt et al. (22), UK	**Study design:** Case report, mixed methods **Type of Intervention:** Macmillan Canter Support provided welfare advice to people with cancer and their careers. In in primary and secondary healthcare settings and participants homes. **Data collection methods/outcomes measured:** welfare benefit claims; user and provider experience, including impact on stress and engaging in daily activities **Quality rating:** High	**Sample size:** *n* = 1,174 **Participants and setting:** patients diagnosed with cancer and their careers, UK. **Dates of data collection**: April 2009–March 2010	**Primary findings:** Welfare benefit claims increased by £70.30 per week (median). **Additional findings:** Participants reported reduced levels of stress and anxiety, and an increase in wellbeing (qualitative data).
Evans and McAteer (23), UK	**Study design:** Quasi-experimental, mixed methods with control (for economic analysis) **Type of Intervention:** Social housing organizations provided debt advice to tenants with rent arears. **Data collection methods/outcomes measured:** changes in rent arears, ROI, service users' evaluation of effectiveness for the debt advice. **Quality rating:** Low	**Sample size:** *n* = 411 (*n* = 92 for intervention, *n* = 319 for control) for economic analysis. For qualitative analysis *n* = 179 for qualitative study **Participants and setting:** Adult social housing tenants in the London area with rent arears. **Dates of data collection:** July 2010–Oct. 2011	**Primary findings:** Landlords gained £122 for every £100 invested, due to reduced arears and associated costs. Average reduction of £360 rent arrears per service user a year after using service. **Additional findings:** Half of service users reported that the service helped them avoid eviction or court proceedings.
Howel et al. (24), UK	**Study design:** Pilot RCT, mixed methods **Type of Intervention:** Local authority provided welfare advice delivered in participants' homes by trained advisors. **Data collection methods/outcomes measured:** Changes in income and economic evaluation using CCA and CUA. Health related QoL (EQ-%D questionnaire), social and physical function. **Quality rating:** Medium	**Sample size:** *n* = 755 (intervention *n* = 381, control *n* = 374) **Participants and setting:** Adults 60 years or over living in socio-economically deprived areas of, NE England. **Dates of data collection:** Over 24-month period, with recruitment between May 2012 and Feb. 2013	**Primary findings:** Only 22% received additional benefits as participants were more affluent than expected (inclusion was based on status of area rather than individual). There was no effect on health outcomes. The intervention was not cost-effective. **Additional findings:** The CUA showed that the intervention was more costly but also more effective than usual care. CCA highlighting that 38% of delivery costs were due to advisors traveling to participants' homes

## Results

Of the nine papers identified, eight described studies conducted in the UK (17–24), and one described a study conducted in the US (16). Four were studies published in peer-reviewed journals (16, 18, 22, 24) and five were non-peer reviewed reports including: a 2015-16 Citizens Advice (CA) report (17), a National Institute of Health & Care Research (NIHR) study report (19), University of Chester digital repository report (20), a study report from the Glasgow Centre for Population Health (21), and a UK-based housing association study report (23). Of the nine studies, seven were mixed methods studies, with five of these being case reports (16, 17, 20–22), two being pilot RCTs (19, 24) and one was a quasi-experimental controlled trial (23). The ninth study was a quantitative quasi-experimental controlled trial (18). Only one study was classified as a HJP intervention (16); four of the studies had welfare advice provided by CA (17–20) or Money Advice, which is an equivalent service based in Scotland (21). One study described welfare advice provided by Macmillan Cancer Support (22), one study described social landlord associations providing welfare advice (23), and in one study welfare advice was provided by local authority welfare department (24). Of the nine studies included, the four peer reviewed studies (16, 18, 22, 24) were of moderate to high quality, according to the JBI quality appraisal checklists. Whereas the non-peer reviewed studies (17, 19–21, 23) were of low to moderate quality (see Appendix).

The single study that reported on a HJP was a study on the impact of civil legal aid services for women experiencing intimate partner violence in the US (16). This small study (*n* = 82) reported that 12 months after initial service use, women had an average increase in income of $5,500, which was driven by significant increases in wages from jobs and child support (private income) and decreases in food stamps (public income). The scheme had a positive return on investment (ROI), as women's overall income increased by $2.41 for every $1 spent on the service. A year after accessing the service, the odds of women being in poverty was approx. 2.5 times lower [reverse odds ratio (rOR) = 0.28] than before service use. This indicated that as well as the financial improvements to service users and government (due to the reduction in public support) the intervention was able to improve the socio-economic status of service users.

The CA 2015/16 financial evaluation (17) reported a ROI of £20.57 for every £1 invested in welfare advice services. This included £10.97 financial increase to service users (through benefits gained, debts written off and consumer problems resolved), £1.52 of savings to government (due to reduction in benefits claimed and health service demand), and £8.08 in wider economic and social benefits (for clients and CA volunteers). Sixty-five to seventy-five percent clients also stated that welfare issues were causing them stress and anxiety, although the impact of service use on these measures was not assessed.

Several of the studies included reviewed the provision of CA welfare advice services in primary or secondary healthcare settings. Woodhead et al., evaluated the cost-effectiveness of co-located welfare services, and the impact of debt advice on mental health and primary healthcare use (18). The study reported a ROI of £15 to service users per £1 of funder investment, with an average financial gain per participant of £2,689 over the 8-month study period. The service also reduced the level of common mental disorders (CMD) diagnoses for specific demographic groups, with both women and black service users having a reduced likelihood of CMD diagnosis compared to controls (rOR = 0.37 and rOR = 0.09, respectively). The authors reported that after 3 months there were no significant changes in GP consultation rates, although this was a shorter evaluation period than the 8 months used for the financial evaluation.

A feasibility RCT to determine the effect of debt counseling in primary care settings on mental health and healthcare use (19) was not able to complete the planned health economics evaluation due to a small trial size (*n* = 61) and high dropout rate over the 12-months of the trial. Although the study did not report cost-effectiveness, we have included this study as it was one of the few studies to aim to complete a full economic evaluation. In addition, descriptive statistics from the health economic dataset indicate that the intervention group did have lower levels of acute admissions and higher levels of community service use for mental healthcare or alcohol/substance misuse than the control group. Further studies are needed to determine if these reported changes indicate significant changes in use of healthcare due to a welfare intervention.

Caiels and Thurston also reported an average of £1,041 financial gains for service users of welfare advice in primary care (20). Although the study did not include a ROI analysis, CA consultancy time per case type was included. A high number (68%) of services users also reported improvements in anxiety and general health in qualitative interviews, but these changes were not corroborated by the self-report questionnaire data (SF-12).

Three of the included studies focused on the provision of legal or welfare advice for specific target groups or welfare issues. The Healthier, Wealthier Children Project (21) provided welfare advice services to pregnant women and families with young children at risk of, or experiencing, child poverty. Welfare advice was provided in healthcare settings with referrals by midwifes and health visitors. The service identified that almost half of service users were entitled to additional financial support and reported an average gain per service user of £3,404. Improvements in wellbeing were reported in qualitative interviews, but not quantified.

Moffatt et al. (22) evaluated a Macmillan Cancer Support funded welfare rights service to cancer patients. The authors reported that 96% of service users had successful welfare benefit claims, which led to a median increased weekly income of £70.30. A full cost evaluation was not completed in this study. Qualitative analysis highlighted that the impact of the intervention had lessened the impact of lost earnings, helped offset the costs associated with cancer, increased the ability to maintain independence, reduced stress and anxiety, and improved wellbeing and quality of life. Although this study did not look to include changes in these factors in their evaluation, these are factors that may have the potential to show a higher level of economic return on this type of intervention.

Evans and McAteer (23) reported that a scheme for social landlords providing debt advice to their tenants who were in debt arears decreased tenants' debt arrears by an average of £360 a year. The scheme had a positive ROI, with landlords recouping £122 for every £100 invested, due to both a reduction in arrears and the costs associated with addressing the debt. Half of participants reported that the service had helped them avoid court appearances or eviction. The threat of eviction is often a traumatic experience to tenants (26) and evictions also have high-cost implications for landlords (27). So, the study demonstrated that provision of welfare legal advice services can have financial benefits to both tenants and landlords, with potential further economic benefits due positive impacts of health and wellbeing of tenants.

Howel et al. (24) was the only paper to include both financial and health measures in their economic analyses. This included a within-trial cost-consequence analysis (CCA) to provide a breakdown and range of individual costs and benefits, and a Cost-Utility Analysis (CUA) that included both cost data and a measure of health-related quality of life (HRQoL) using the EQ-5D questionnaire. Although the provision of domiciliary advice to older people in socio-economically deprived areas was not cost effective in this trial, the study highlighted several issues that could be used as guidance for future studies. By targeting geographical areas rather than individuals with socio-economic inequalities, participants turned out to be more affluent than anticipated and therefore less eligible for the welfare service, indicating the need for more tailored and targeted interventions. The CUA showed that the intervention was more costly but also more effective than usual care, with the CCA highlighting that 38% of delivery costs were due to advisors traveling to participants' homes. This indicates that further work is required to explore he potential cost-effectiveness of services provided outside CA or healthcare settings. This may include online or group-based services or co-location with other multi-disciplinary centers already being used by potential service users, such as social/community centers or food banks.

## Discussion

The aim of this review was to determine the current evidence of the cost-effectiveness of legal and welfare rights services. Only nine studies were identified, including four peer-reviewed studies (of medium-high quality) and five non-peer reviewed reports (of low-medium quality).

Eight of the included studies reported financial improvement to services users, with the ninth study unable to report due to low recruitment. However, only three of the papers reported on cost-effectiveness of interventions or included an assessment on the financial impact on stakeholders other than service users. Although this is in line with the studies' perspective, it is perhaps surprising that ROI was not more consistently investigated, considering that the focus of the studies was financial gain due to service provision.

In addition, few studies looked to determine the wider impact of the interventions on health or health service use. Although many of the studies used qualitative interviews to investigate changes in mental and physical health, only three studies used quantitative measures and one study used a health economic measure (CUA) to evaluate the impact of the interventions on participants' health. As discussed in the introduction, a significant amount of primary and secondary healthcare usage is reported to be driven by non-health socio-legal issues. However only one study reported on changes in healthcare usage (18). No changes were reported after 3 months in the study, although this was acknowledged as a rather short time period for follow-up. As the socio-economic problems that are addressed by legal and welfare programs often have adverse impacts on mental and physical health, it seems reasonable to conclude that health measures should be included in legal and welfare service evaluations.

The Legal Aid, Sentencing and Punishment of Offenders (LASPO) Act of 2012 is acknowledged to have significantly restricted legal aid funding in the UK (28). It is perhaps not surprising that many of the studies identified in this review were provided by charities such as CA. CA provides similar welfare advice to HJP, including providing advocacy to some clients, and has continued to receive funding from government and local councils post-2012. However, the search included pre-2012 studies and US based studies, where HJP is well-established (15). As only one HJP specific study, and only a total of four peer-reviewed welfare intervention studies with health economic evaluations were identified in this review, it indicates that there is a lack of economic evaluations of these services.

We propose that developing effective methods to measure the impact of HJP and similar interventions may be difficult due to the following reasons. These types of interventions are often used to treat diverse populations or interventions, where outcomes can vary significantly across different population groups or intervention types. There may be barriers to information sharing between healthcare organizations and legal partners, which was highlighted in a recent study (29). There are also considerable differences in the US and UK healthcare systems, the two countries with the main utilization of health justice interventions (15), which may mean it is difficult to compare the impact of interventions. The requirement to develop a more standardized approach to evaluating legal and welfare studies has been highlighted (30). A more consistent quantitative approach to intervention assessments would also give the ability to provide a consistent health economic assessment of these types of interventions.

In conclusion, this rapid literature review highlights the current evidence gap in what we know about the potential cost-effectiveness to society of enabling people to have adequate access to welfare advice and specialist free legal advice and representation. Based on the geographical location of reviewed articles we propose an initial UK/US focused interdisciplinary research agenda between health economics and legal-health services to address this research gap, with the intention to extend this to a more international research agenda in future years.

## Author contributions

RG, HG, and RT: review conceptualization. RG: database searches, article assessments, paper reviews, paper quality assessments, and writing review. HG and RT: editing review and supervision. All authors have read and agreed to the published version of the manuscript.

## Conflict of interest

The authors declare that the research was conducted in the absence of any commercial or financial relationships that could be construed as a potential conflict of interest.

## Publisher's note

All claims expressed in this article are solely those of the authors and do not necessarily represent those of their affiliated organizations, or those of the publisher, the editors and the reviewers. Any product that may be evaluated in this article, or claim that may be made by its manufacturer, is not guaranteed or endorsed by the publisher.
