# Supplementary material for: Health economics of health justice partnerships: A rapid review of the economic returns to society of promoting access to legal advice
Source: Front Public Health. 2022 Nov 15;10:1009964. doi: 10.3389/fpubh.2022.1009964 (PMC9705517; doi:10.3389/fpubh.2022.1009964)
Supplement: Supplementary file 1 [file Table_1.DOCX]

Supplementary Material: for appendix

# Supplementary Tables

**Appendix: Quality Apraisal Tables, using JBI methodology**

**Table 1: JBI Case Report Quality Appraisal Checklist**

| **CITATION** | **16**  **Teuful et al.**  **(2021)** | **20**  **Caiels & Thurston**  **(2005)** | **21**  **Naven et al.**  **(2012)** | **22**  **Moffatt et al., (2012)** |
| --- | --- | --- | --- | --- |
| Q1. Were patient’s demographic characteristics clearly described? | Yes | Unsure | No | Yes |
| Q2. Was the patient’s history clearly described and presented as a timeline? | No | No | No | Unsure |
| Q3. Was the current clinical condintion of the patient or presentation clearly described? | Unsure | No | No | Yes |
| Q4. Were diagnostic tests or assessment methods and the results clearly described? | Yes | Yes | Yes | Yes |
| Q5. Was the intervention(s) or treatment procedures(s) clearly described? | Unsure | Unsure | Unsure | Yes |
| Q6. Was the post-intervention clinical condition clearly described? | Yes | Yes | Yes | Yes |
| Q7. Were adverse events (harms) or unanticipated events identified and described? | No | No | No | Unsure |
| Q8. Does the case report provide takeaway lessons? | Yes | No | Yes | Yes |

**Table2: JBI RTC Quality Appraisal Checklist**

| **CITATION** | **19**  **Gabbay et al., (2017)** | **24**  **Howel et al.**  **(2019)** |
| --- | --- | --- |
| Q1. Was true randomization used for assignment of participants to treatment groups? | Yes | No |
| Q2. Was allocation to treatment groups concealed? | Yes | Unsure |
| Q3. Were treatment groups similar at the baseline? | Yes | Yes |
| Q4. Were participants blind to treatment assignment? | Yes | No |
| Q5. Were those delivering treatment blind to treatment assignment? | No | No |
| Q6. Were outcomes assessors blind to treatment assignment? | No | Yes |
| Q7. Were treatment groups treated identically other than the intervention of interest? | Yes | Yes |
| Q8. Was follow up complete and if not, were differences between groups in terms of their follow up adequately described and analyzed? | Unsure | Unsure |
| Q9. Were participants analyzed in the groups to which they were randomized? | Yes | Yes |
| Q10. Were outcomes measured in the same way for treatment groups? | Yes | Yes |
| Q11. Were outcomes measured in a reliable way? | Yes | Yes |
| Q12. Was appropriate statistical analysis used? | Yes | Yes |
| Q13. Was the trial design appropriate, and any deviations from the standard RCT design (individual randomization, parallel groups) accounted for in the conduct and analysis of the trial? | Yes | Yes |

**Table 3. JBI Quasi-Experimental Quality Appraisal Checklist**

| **CITATION** | **18**  **Woodhead et al.**  **(2017)** | **23**  **Evans & McAteer**  **(2011)** |
| --- | --- | --- |
| Q1. Is it clear in the study what is “cause” and what is “effect? | Yes | Yes |
| Q2. Where the participants included in any comparisons similar? | Yes | Unsure |
| Q3. Were participants included in comparisons receiving similar treatment/car, other than intervention/exposure? | Yes | Unsure |
| Q4. Was there a control group? | Yes | Yes |
| Q5. Were there multiple measurements of outcome pre- and post- intervention  /exposure? | Yes | Unsure |
| Q6 Was follow up complete, or if not were differences between groups adequately described and analysed? | Unsure | Unsure |
| Q7 Were the outcomes of participants included in any comparisons measured in the same way? | Yes | Unsure |
| Q8 Were outcomes measured in a reliable way? | Yes | Unsure |
| Q9 Was appropriate statistical analysis used? | Yes | No |

**Table 4: JBI Economic Evaluations** **Quality Appraisal Checklist**

| **CITATION** | **17**  **Citizans Advice**  **(2016)** |
| --- | --- |
| Q1 Is there a well-defined question? | Yes |
| Q2 Is there comprehensive description of alternatives? | No |
| Q3 Are all important and relevant costs and outcomes for each alternative identified? | Unsure |
| Q4 Has clinical effectiveness been established? | Yes |
| Q5 Are costs and outcomes measured accurately? | Unsure |
| Q6 Are costs and outcomes valued credibly? | Unsure |
| Q7 Are costs and outcomes adjusted for differential timing? | No |
| Q8 Is there an incremental analysis of costs and consequences? | No |
| Q9 Were sensitivity analyses conducted to investigate uncertainty in estimates of cost or consequences? | No |
| Q10 Do study results include all issues of concern to users? | Unsure |
| Q11 Are the results generalizable to the setting of interest in the review? | Yes |

**
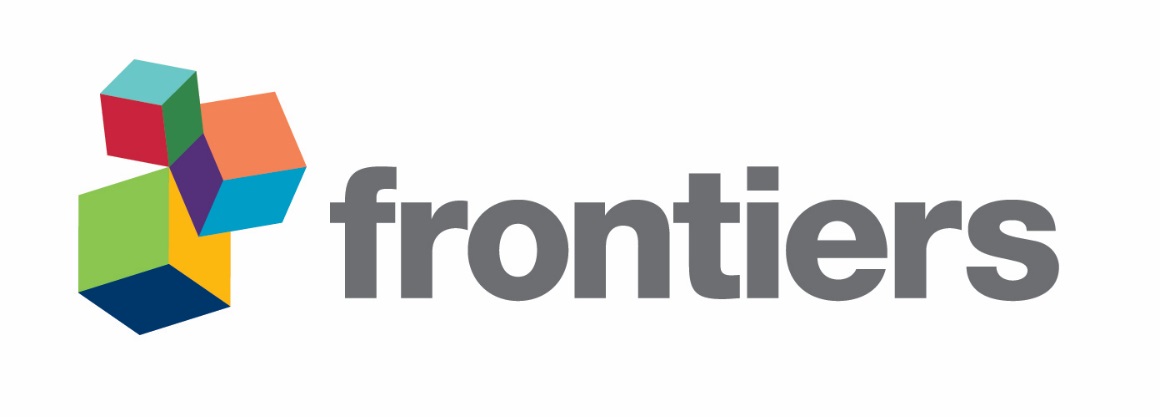
**
